# Supplementary material for: Quantifying Heart Rate Changes After Delta-9-Tetrahydrocannabinol Administration Using a PBPK-PD Model in Healthy Adults
Source: Pharmaceutics. 2025 Feb 12;17(2):237. doi: 10.3390/pharmaceutics17020237 (PMC11858910; doi:10.3390/pharmaceutics17020237)
Supplement: Supplementary file 1 [file pharmaceutics-17-00237-s001.zip › pharmaceutics-3439871-supplementary.pdf]

SUPPLEMENTARY MATERIALS

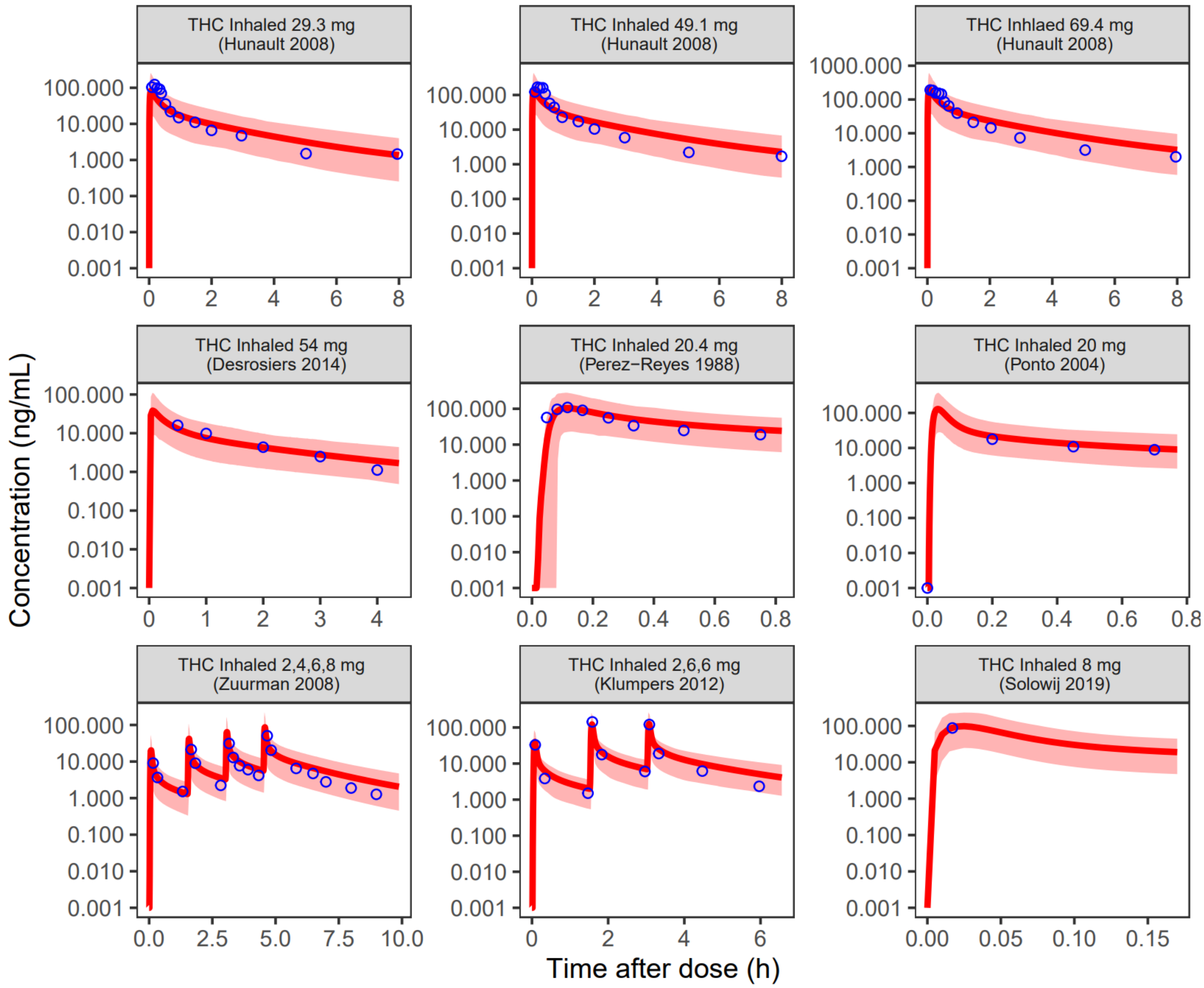

Figure S1. Observed and PBPK model-predicted THC plasma concentrations following inhaled THC administrations. The red shaded areas represent the 5th to 95th percentiles of predicted values. The red lines and blue circles represent the mean predicted values and observed concentrations, respectively.

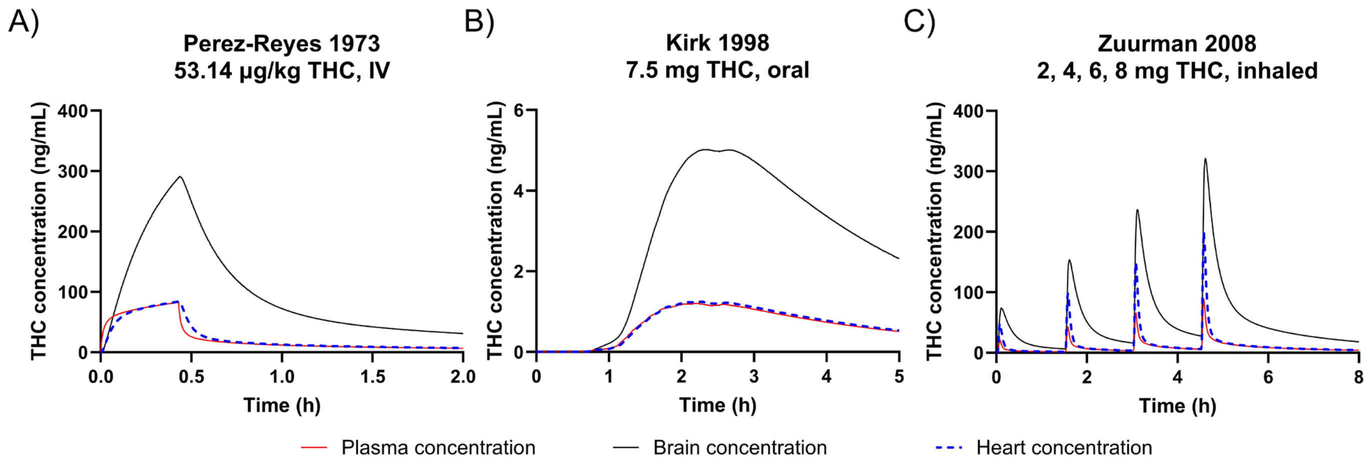

Figure S2. Predicted THC plasma, brain, and heart concentration after **(A)** intravenous (IV), **(B)** oral, and **(C)** inhaled THC administration.

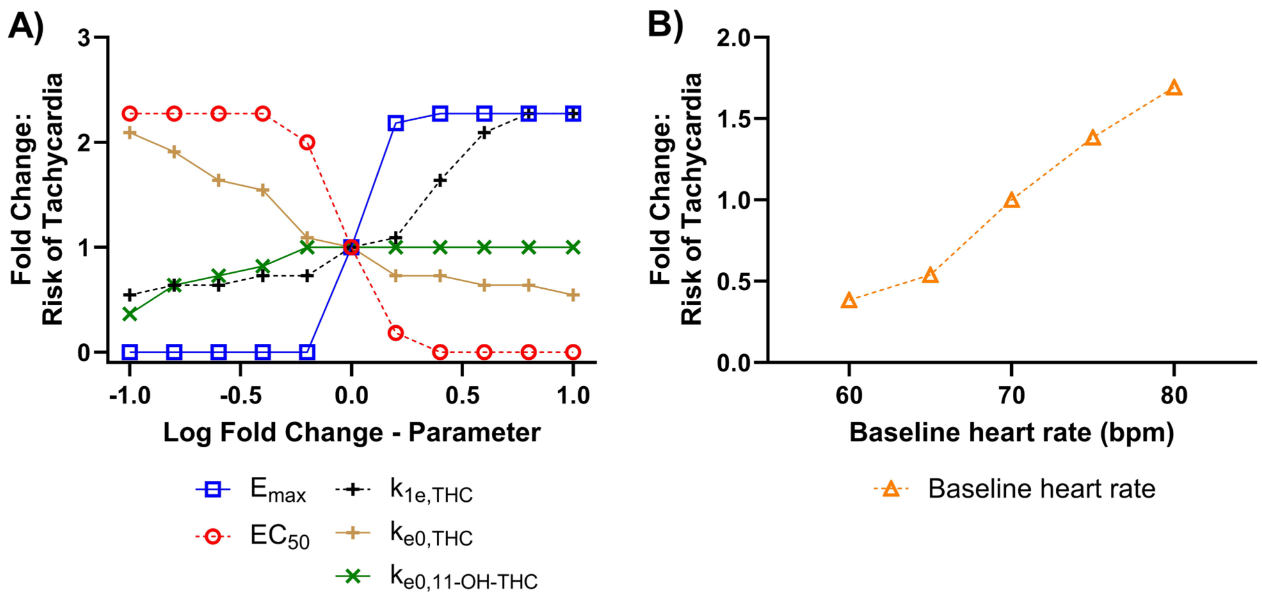

Figure S3. Sensitivity analyses assessing the impact of various PD model parameters on the risk of tachycardia. (**A**) Parameters from the effect compartment and E_{max} model and (**B**) baseline heart rate. E_{max}, the maximum increase fraction from the baseline heart rate; EC_{50}, concentration in the effect compartment required to obtain 50% of the maximum change; k_{1e}, rate constants for transport through the transfer compartment; k_{e0}, rate constants for loss from the effect compartment.

Table S1. PBPK-PD predicted and observed heart rates or percentage changes in heart rate from baseline following THC administration in healthy adults.

Formulation	Trial	PD Group [a]	PK Group [b]	Dose regimen	Absorption Parameters	Observed Peak Heart Rate (bpm)	Predicted Peak Heart Rate (bpm)	Peak Heart Rate Predicted: Observed
**THC**								
IV	Perez-Reyes 1973 [1]	Modeling	/	53.14 μg/kg	/	55.7 [c]	54.2 [c]	0.97
	Perez-Reyes 1972 [2]	Validation	/	3.1mg	/	50.3 [c]	48.8 [c]	0.97
	Mathew 1999 [3]	Validation	/	0.15 mg/kg	/	92.3	90.6	0.98
		Validation	/	0.25 mg/kg	/	97.5	102.7	1.05
Oral	Kirk 1998 [4]	Verification	/	7.5 mg	f_a = 0.45, k_a = 0.7	78.9	78.4	0.99
		Verification	/	15 mg	f_a = 0.45, k_a = 0.7	86.0	82.2	0.96
	Abboud 1976 [5]	Validation	/	10 mg	f_a = 0.45, k_a = 0.7	10.0 [c]	12.2 [c]	1.22
	McDonald 2003 [6]	Validation	/	7.5 mg	f_a = 0.45, k_a = 0.7	73.2	79.2	1.08
		Validation	/	15 mg	f_a = 0.45, k_a = 0.7	77.1	84.1	1.09
	Klooker 2011 [7]	Validation	/	5 mg	f_a = 0.45, k_a = 0.7	74.2	68.3	0.92
		Validation	/	10 mg	f_a = 0.45, k_a = 0.7	72.3	74.9	1.04
	Murray 2022 [8]	Validation	/	7.5 mg	f_a = 0.45, k_a = 0.7	71.8	66.3	0.92
		Validation	/	15 mg	f_a = 0.45, k_a = 0.7	78.5	69.8	0.89
	Shabani 2023 [9]	Validation	/	10 mg	f_a = 0.45, k_a = 0.7	70.8	75.2	1.06
Inhaled	Zuurman 2008 [10]	Verification	Type 2	2, 4, 6, 8 mg [d]	F_{inh} = 0.6, lung k_a = 200	95.8	99.0	1.03
	Hunault 2009 [11]	Validation	Type 1	29.3 mg [e]	F_{inh} = 0.22, lung k_a = 12	122.1	117.0	0.96
		Validation	Type 1	49.1 mg [e]	F_{inh} = 0.22, lung k_a = 12	125.5	128.1	1.02
		Validation	Type 1	69.4 mg [e]	F_{inh} = 0.22, lung k_a = 12	129.7	129.0	0.99
	Perez-Reyes 1988 [12]	Validation	Type 2	20.4 mg [d]	F_{inh} = 0.5, lung k_a = 12	49.2 [c]	49.9 [c]	1.01
	Ponto 2004 [13]	Validation	Type 2	20 mg [d]	F_{inh} = 0.22, lung k_a = 200	103.0	97.7	0.95
	Klumpers 2012 [14]	Validation	Type 2	2, 6, 6 mg [d]	F_{inh} = 0.9, lung k_a = 200	89.2	87.9	0.99
	Desrosiers 2015 [15]	Validation	Type 2	54 mg [d]	F_{inh} = 0.05, lung k_a = 12	106.3	91.2	0.86
	Solowij 2019 [16]	Validation	Type 2	8 mg [d]	F_{inh} = 0.4, lung k_a = 200	108.6	102.6	0.94
	Heishman 1989 [17]	Validation	Type 3	12 mg	F_{inh} = 0.07, lung k_a = 200	99.4	94.3	0.95
		Validation	Type 3	21 mg	F_{inh} = 0.07, lung k_a = 200	109.2	107.7	0.99
	Low 1973 [18]	Validation	Type 3	4.8 mg	F_{inh} = 0.6, lung k_a = 200	89.9	90.8	1.01
		Validation	Type 3	9.1 mg	F_{inh} = 0.6, lung k_a = 200	102.2	99.8	0.98
	Tashkin 1973 [19]	Validation	Type 3	0.07 mg/kg	F_{inh} = 0.6, lung k_a = 200	40.9 [c]	42.4 [c]	1.04

Formulation	Trial	PD Group [a]	PK Group [b]	Dose regimen	Absorption Parameters	Observed Peak Heart Rate (bpm)	Predicted Peak Heart Rate (bpm)	Peak Heart Rate Predicted: Observed
Inhaled	Tashkin 1973 [19]	Validation	Type 3	0.14 mg/kg	F_{inh} = 0.6, lung k_a = 200	55.7 [c]	57.5 [c]	1.03
	Ashton 1981 [20]	Validation	Type 3	2.5 mg	F_{inh} = 0.22, lung k_a = 12	82.9	82.2	0.99
		Validation	Type 3	10 mg	F_{inh} = 0.22, lung k_a = 12	100.3	98.1	0.98
	Fant 1998 [21]	Validation	Type 3	15.6 mg	F_{inh} = 0.1, lung k_a = 200	97.4	92.6	0.95
		Validation	Type 3	25.1 mg	F_{inh} = 0.1, lung k_a = 200	98.1	95.9	0.98
	Tashkin 1977 [22]	Validation	Type 3	20 mg	F_{inh} = 0.22, lung k_a = 200	51.7 [c]	53.2 [c]	1.03
	Sulkowski 1977 [23]	Validation	Type 3	10 mg	F_{inh} = 0.9, lung k_a = 200	132.4	101.3	0.77
	Dalton 1976 [24]	Validation	Type 3	25 μg/kg	F_{inh} = 0.9, lung k_a = 200	99.4	93.7	0.94
	Evans 1976 [25]	Validation	Type 3	50 μg/kg	F_{inh} = 0.9, lung k_a = 200	91.0	78.3	0.86
	Zacny 1989 [26]	Validation	Type 3	11.05 mg	F_{inh} = 0.1, lung k_a = 200	85.4	85.6	1.00
	Chait 1985 [27]	Validation	Type 3	29 mg	F_{inh} = 0.5, lung k_a = 12	103.0	119.3	1.16
	Karniol 1973 [28]	Validation	Type 3	20 mg	F_{inh} = 0.5, lung k_a = 200	138.4	136.0	0.98
	Clark 1974 [29]	Validation	Type 3	9 mg	F_{inh} = 0.4, lung k_a = 12	88.0	84.1	0.96
	Zuurman 2010 [30]	Validation	Type 3	2, 4, 6, 6 mg	F_{inh} = 0.5, lung k_a = 12	88.7	82.6	0.93
**11-OH-THC**								
IV	Perez-Reyes 1973 [1]	Modeling	/	46.99 μg/kg	/	63.1 [c]	66.7 [c]	1.06
	Perez-Reyes 1972 [2]	Validation	/	2.27mg	/	53.0 [c]	61.7 [c]	1.16

bpm, beats per minute; IV, intravenous; /, not applied; F_{inh}, fraction of drug absorbed from the inhalation; lung k_a, first-order absorption rate constant of lung absorption.

[a] Model: dataset was used for developing the PD model; Verify: dataset was used for PD model verification; Validate: PD model validation dataset includes studies using IV, oral, and inhaled THC administration.

[b] Type 1:clinical trials with THC concentration-time profiles included in our previous THC PBPK study [31]; Type 2: clinical trials with THC concentration-time profiles not included in our previous study; Type 3: clinical trials provided only dosing information but no THC concentration-time profiles.

[c] The percentage change in heart rate from baseline was either reported or simulated.

[d] The THC concentration-time profiles were provided in the literature.

[e] The THC concentration-time profiles were proved in Hunault 2008 [32].

Lua code for the THC heart rate PD model, return heart rate (bpm) or heart rate change percentile (%)

```
--Author: Lixuan Qian, Zhu Zhou

--Created on: 2024-07-12



--This custom Lua model can only run in Simcyp™ PBPK Simulator (version 22), a higher version may report
error messages (which can be easily solved).

--Run in Custom Lua Models; does not work in PD Basic 1 – PD Custom

function initCustomOdeStates()
  local states = simcyp.CustomOdeStates()
  states:add("THC Effect Comp", simcyp.unit.Unknown, 0)
  states:add("11-OH-THC Effect Comp", simcyp.unit.Unknown, 0)
  states:add("Heart Rate", simcyp.unit.Unknown, 0)
  return states
end

function initCustomIndividualParameters(individual)
  local params = simcyp.CustomParameters()
  return params
end

function initCustomCompoundParameters(individual, compounds)
  local params = simcyp.CustomParameters()
  params:add("k1e1", sc:sampleRandomDistribution(sc.LOGNORMAL_CV, 4, 0))
  params:add("ke01", sc:sampleRandomDistribution(sc.LOGNORMAL_CV, 6.5, 0))
  params:add("ke02", sc:sampleRandomDistribution(sc.LOGNORMAL_CV, 15, 0))
  params:add("Emax", sc:sampleRandomDistribution(sc.TRUNCATED_NORMAL_CV, 105, 32))
  params:add("EC50", sc:sampleRandomDistribution(sc.TRUNCATED_NORMAL_CV, 0.15, 79))
  params:add("Baseline", sc:sampleRandomDistribution(sc.TRUNCATED_NORMAL_CV, 64.9, 0))
  return params
end

function initCustomOdeEventTimes(simulationDuration)
  eventTimes = {}
  step = 0.005
  for i=1, math.floor(simulationDuration / step) do
    eventTimes[i] = (i-1) * step
  end
    return eventTimes
```

```lua
function handleCustomOdeEventStep(eventIndex, t, su, states, individual, compounds, isInhibition)

  local C_THCE, C_OHTHCE, EC50, Emax, Baseline, HR
  C_THCE   = states[1]["THC Effect Comp"]
  C_OHTHCE   = states[1]["11-OH-THC Effect Comp"]
  Emax = compounds[sc.SUBSTRATE].Emax
  EC50 = compounds[sc.SUBSTRATE].EC50
  Baseline = compounds[sc.SUBSTRATE].Baseline
--For heart rate count (bpm)
   HR = (Emax * (su[C_THCE] + su[C_OHTHCE])/ (EC50 + (su[C_THCE] + su[C_OHTHCE]))/100+1) * Baseline

--For heart rate change percentile (%)

--HR = Emax * (su[C_THCE] + su[C_OHTHCE])/ (EC50 + (su[C_THCE] + su[C_OHTHCE])) + Baseline
  su[states[1]["Heart Rate"]] = HR
end

function handleCustomOdeRateStep(t, su, gu, states, individual, compounds, isInhibition)

  local k1e1, ke01, ke02, C_THC, C_OHTHC, C_THCE, C_OHTHCE
  k1e1 = compounds[sc.SUBSTRATE].k1e1
  ke01 = compounds[sc.SUBSTRATE].ke01
  ke02 = compounds[sc.SUBSTRATE].ke02
  C_THC       = su[states[0]["Substrate heart concentration"]]
  C_OHTHC     = su[states[0]["Sub Pri Metabolite1 heart concentration"]]
  C_THCE   = su[states[1]["THC Effect Comp"]]
  C_OHTHCE   = su[states[1]["11-OH-THC Effect Comp"]]
  gu[states[1]["THC Effect Comp"]] = k1e1 * C_THC – ke01 * C_THCE
  gu[states[1]["11-OH-THC Effect Comp"]] = ke02 * (C_OHTHC - C_OHTHCE)
  gu[states[1]["Heart Rate"]] = 0
  end

--end of the Lua code
```

References

1. Perez-Reyes, M., M. C. Timmons, M. A. Lipton, H. D. Christensen, K. H. Davis, and M. E. Wall. "A Comparison of the Pharmacological Activity of Delta 9-Tetrahydrocannabinol and Its Monohydroxylated Metabolites in Man." *Experientia* 29, no. 8 (1973): 1009-10.
2. Perez-Reyes, M., M. C. Timmons, M. A. Lipton, K. H. Davis, and M. E. Wall. "Intravenous Injection in Man of 9 - Tetrahydrocannabinol and 11-Oh- 9 -Tetrahydrocannabinol." *Science* 177, no. 4049 (1972): 633-5.
3. Mathew, R. J., W. H. Wilson, N. Y. Chiu, T. G. Turkington, T. R. Degrado, and R. E. Coleman. "Regional Cerebral Blood Flow and Depersonalization after Tetrahydrocannabinol Administration." *Acta Psychiatr Scand* 100, no. 1 (1999): 67-75.
4. Kirk, J. M., P. Doty, and H. De Wit. "Effects of Expectancies on Subjective Responses to Oral Delta9-Tetrahydrocannabinol." *Pharmacol Biochem Behav* 59, no. 2 (1998): 287-93.
5. Abboud, R. T., and H. D. Sanders. "Effect of Oral Administration of Delta-Tetrahydrocannabinol on Airway Mechanics in Normal and Asthmatic Subjects." *Chest* 70, no. 4 (1976): 480-5.
6. McDonald, J., L. Schleifer, J. B. Richards, and H. de Wit. "Effects of Thc on Behavioral Measures of Impulsivity in Humans." *Neuropsychopharmacology* 28, no. 7 (2003): 1356-65.
7. Klooker, T. K., K. E. Leliefeld, R. M. Van Den Wijngaard, and G. E. Boeckxstaens. "The Cannabinoid Receptor Agonist Delta-9-Tetrahydrocannabinol Does Not Affect Visceral Sensitivity to Rectal Distension in Healthy Volunteers and Ibs Patients." *Neurogastroenterol Motil* 23, no. 1 (2011): 30-5, e2.
8. Murray, C. H., Z. Huang, R. Lee, and H. de Wit. "Adolescents Are More Sensitive Than Adults to Acute Behavioral and Cognitive Effects of Thc." *Neuropsychopharmacology* 47, no. 7 (2022): 1331-38.
9. Shabani, M., M. Ilaghi, R. Naderi, and M. Razavinasab. "The Hyperexcitability of Laterodorsal Tegmentum Cholinergic Neurons Accompanies Adverse Behavioral and Cognitive Outcomes of Prenatal Stress." *Sci Rep* 13, no. 1 (2023): 6011.
10. Zuurman, L., C. Roy, R. C. Schoemaker, A. Hazekamp, J. den Hartigh, J. C. Bender, R. Verpoorte, J. L. Pinquier, A. F. Cohen, and J. M. van Gerven. "Effect of Intrapulmonary Tetrahydrocannabinol Administration in Humans." *J Psychopharmacol* 22, no. 7 (2008): 707-16.
11. Hunault, C. C., T. T. Mensinga, K. B. Böcker, C. M. Schipper, M. Kruidenier, M. E. Leenders, I. de Vries, and J. Meulenbelt. "Cognitive and Psychomotor Effects in Males after Smoking a Combination of Tobacco and Cannabis Containing up to 69 Mg Delta-9-Tetrahydrocannabinol (Thc)." *Psychopharmacology (Berl)* 204, no. 1 (2009): 85-94.
12. Perez-Reyes, M., R. E. Hicks, J. Bumberry, A. R. Jeffcoat, and C. E. Cook. "Interaction between Marihuana and Ethanol: Effects on Psychomotor Performance." *Alcohol Clin Exp Res* 12, no. 2 (1988): 268-76.
13. Ponto, L. L., D. S. O'Leary, J. Koeppel, R. I. Block, G. L. Watkins, J. C. Richmond, C. A. Ward, D. A. Clermont, B. A. Schmitt, and R. D. Hichwa. "Effect of Acute Marijuana on Cardiovascular Function and Central Nervous System Pharmacokinetics of [(15)O]Water: Effect in Occasional and Chronic Users." *J Clin Pharmacol* 44, no. 7 (2004): 751-66.
14. Klumpers, L. E., D. M. Cole, N. Khalili-Mahani, R. P. Soeter, E. T. Te Beek, S. A. Rombouts, and J. M. van Gerven. "Manipulating Brain Connectivity with Δ^9-Tetrahydrocannabinol: A Pharmacological Resting State Fmri Study." *Neuroimage* 63, no. 3 (2012): 1701-11.
15. Desrosiers, N. A., J. G. Ramaekers, E. Chauchard, D. A. Gorelick, and M. A. Huestis. "Smoked Cannabis' Psychomotor and Neurocognitive Effects in Occasional and Frequent Smokers." *J Anal Toxicol* 39, no. 4 (2015): 251-61.
16. Solowij, N., S. Broyd, L. M. Greenwood, H. van Hell, D. Martelozzo, K. Rueb, J. Todd, Z. Liu, P. Galettis, J. Martin, R. Murray, A. Jones, P. T. Michie, and R. Croft. "A Randomised Controlled Trial of Vaporised Δ(9)-Tetrahydrocannabinol and Cannabidiol Alone and in Combination in Frequent and Infrequent Cannabis Users: Acute Intoxication Effects." *Eur Arch Psychiatry Clin Neurosci* 269, no. 1 (2019): 17-35.
17. Heishman, S. J., M. L. Stitzer, and J. E. Yingling. "Effects of Tetrahydrocannabinol Content on Marijuana Smoking Behavior, Subjective Reports, and Performance." *Pharmacol Biochem Behav* 34, no. 1 (1989): 173-9.
18. Low, M. D., H. Klonoff, and A. Marcus. "The Neurophysiological Basis of the Marijuana Experience." *Can Med Assoc J* 108, no. 2 (1973): 157-65.
19. Tashkin, D. P., B. J. Shapiro, and I. M. Frank. "Acute Pulmonary Physiologic Effects of Smoked Marijuana and Oral (Delta)9 - Tetrahydrocannabinol in Healthy Young Men." *N Engl J Med* 289, no. 7 (1973): 336-41.
20. Ashton, H., J. Golding, V. R. Marsh, J. E. Millman, and J. W. Thompson. "The Seed and the Soil: Effect of Dosage, Personality

and Starting State on the Response to Delta 9 Tetrahydrocannabinol in Man." *Br J Clin Pharmacol* 12, no. 5 (1981): 705-20.

21. Fant, R. V., S. J. Heishman, E. B. Bunker, and W. B. Pickworth. "Acute and Residual Effects of Marijuana in Humans." *Pharmacol Biochem Behav* 60, no. 4 (1998): 777-84.
22. Tashkin, D. P., S. Reiss, B. J. Shapiro, B. Calvarese, J. L. Olsen, and J. W. Lodge. "Bronchial Effects of Aerosolized Delta 9-Tetrahydrocannabinol in Healthy and Asthmatic Subjects." *Am Rev Respir Dis* 115, no. 1 (1977): 57-65.
23. Sulkowski, A., L. Vachon, and E. S. Rich, Jr. "Propranolol Effects on Acute Marihuana Intoxication in Man." *Psychopharmacology (Berl)* 52, no. 1 (1977): 47-53.
24. Dalton, W. S., R. Martz, L. Lemberger, B. E. Rodda, and R. B. Forney. "Influence of Cannabidiol on Delta-9-Tetrahydrocannabinol Effects." *Clin Pharmacol Ther* 19, no. 3 (1976): 300-9.
25. Evans, M. A., R. Martz, B. E. Rodda, L. Lemberger, and R. B. Forney. "Effects of Marihuana-Dextroamphetamine Combination." *Clin Pharmacol Ther* 20, no. 3 (1976): 350-8.
26. Zacny, J. P., and L. D. Chait. "Breathhold Duration and Response to Marijuana Smoke." *Pharmacol Biochem Behav* 33, no. 2 (1989): 481-4.
27. Chait, L. D., M. W. Fischman, and C. R. Schuster. "'Hangover' Effects the Morning after Marijuana Smoking." *Drug Alcohol Depend* 15, no. 3 (1985): 229-38.
28. Karniol, I. G., and E. A. Carlini. "Comparative Studies in Man and in Laboratory Animals on 8 - and 9 -Trans-Tetrahydrocannabinol." *Pharmacology* 9, no. 2 (1973): 115-26.
29. Clark, S. C., C. Greene, G. W. Karr, K. L. MacCannell, and S. L. Milstein. "Cardiovascular Effects of Marihuana in Man." *Can J Physiol Pharmacol* 52, no. 3 (1974): 706-19.
30. Zuurman, L., C. Roy, R. C. Schoemaker, A. Amatsaleh, L. Guimaeres, J. L. Pinquier, A. F. Cohen, and J. M. van Gerven. "Inhibition of Thc-Induced Effects on the Central Nervous System and Heart Rate by a Novel Cb1 Receptor Antagonist Ave1625." *J Psychopharmacol* 24, no. 3 (2010): 363-71.
31. Qian, L., T. Zhang, J. Dinh, M. F. Paine, and Z. Zhou. "Physiologically Based Pharmacokinetic Modeling of Cannabidiol, Delta-9-Tetrahydrocannabinol, and Their Metabolites in Healthy Adults after Administration by Multiple Routes." *Clin Transl Sci* 18, no. 1 (2025): e70119.
32. Hunault, C. C., T. T. Mensinga, I. de Vries, H. H. Kelholt-Dijkman, J. Hoek, M. Kruidenier, M. E. Leenders, and J. Meulenbelt. "Delta-9-Tetrahydrocannabinol (Thc) Serum Concentrations and Pharmacological Effects in Males after Smoking a Combination of Tobacco and Cannabis Containing up to 69 Mg Thc." *Psychopharmacology (Berl)* 201, no. 2 (2008): 171-81.
